# Relationships of diabetes self‐care behaviours to glycaemic control in adults with type 2 diabetes and comorbid heart failure

**DOI:** 10.1002/nop2.517

**Published:** 2020-06-15

**Authors:** Fekadu Aga, Sandra B. Dunbar, Tedla Kebede, Melinda Kay Higgins, Rebecca Gary

**Affiliations:** ^1^ School of Nursing & Midwifery College of Health Sciences Addis Ababa University Addis Ababa Ethiopia; ^2^ Nell Hodgson Woodruff School of Nursing Emory University Atlanta GA USA; ^3^ Diabetes & Endocrinology Unit Department of Internal Medicine School of Medicine College of Health Sciences Tikur Anbessa Specialized Hospital Addis Ababa University Addis Ababa Ethiopia

**Keywords:** chronic illness, heart disease, nursing, self‐care, type 2 diabetes

## Abstract

**Aim:**

To describe the relationship between diabetes self‐care behaviours and glycaemic control in patients with type 2 diabetes and comorbid heart failure.

**Design:**

A cross‐sectional, correlational study.

**Method:**

A secondary analysis of 180 participants’ baseline data from a clinical trial that tested a 6‐month integrated self‐care intervention was performed. Correlational and hierarchical linear regression analysis was used to assess the relationships between diabetes self‐care behaviours and glycaemic control.

**Result:**

The Summary of Diabetes Self‐Care Activities general diet and Summary of Diabetes Self‐Care Activities exercise were negatively associated with glycated haemoglobin (HbA1c), while Summary of Diabetes Self‐Care Activities specific diet was positively associated. Diabetic end‐organ failure, taking insulin only and taking both oral antiglycaemic and insulin, predicted higher HbA1c and fasting blood glucose. African American race and dyslipidaemia predicted higher HbA1c while taking higher total daily medication predicted higher fasting blood glucose. Longer years lived with heart failure, lower ventricular ejection fraction and exposure to chemotherapy predicted lower fasting blood glucose.

## 1 INTRODUCTION

Type 2 diabetes mellitus (T2D) is a leading cause of morbidity and mortality worldwide (Zimmet & Alberti, 2016). With the contribution of population growth and ageing, the number of adults with diabetes mellitus (DM) has almost quadrupled during the past two decades (NCD Risk Factor Collaboration [NCD‐RisC, 2016]). The International Diabetes Federation's (IDF) projection indicates that the prevalence of DM in adults (20–79 years) will rise from 425 million (8.8%) in 2017 to 629 million (9.9%) in 2045 worldwide (IDF, 2017). Of this number, over 90% are persons with T2D (Holman, Young, & Gadsby, 2015; Xu et al., 2018). In the United States, T2D is currently the seventh leading cause of death (CDC, 2017).

### 1.1 Background

Individuals with T2D have increased risk for developing cardiovascular disease (CVD) including myocardial infarction, heart failure (HF) and stroke (Peneni & Luscher, 2017). Heart failure was reported as the most common initial manifestation of CVD followed by peripheral arterial disease (Shah et al., 2015). In people with T2D worldwide, the prevalence of HF is 14.9% (Einarson, Acs, Ludwig, & Panton, 2018), which is higher than the 1%–12% in the general population (Roger, 2013). Individuals with T2D and comorbid HF have increased physical limitations, poorer survival rates and quality of life (Bauduceau, Floch, Halimi, Verny, & Doucet, 2018). The person's ability to perform physical activity progressively decrease as the severity of HF advances from the New York Heart Association (NYHA) class II to IV (Kemp & Conte, 2012).

Hyperglycaemia is an important risk factor for CVD events and all‐cause mortality in people with T2D (Afsharian et al., 2016; Takao, Suka, Yangisawa, & Iwamoto, 2017). Evidence concerning the effect of intensive glycaemic control using antidiabetic agents on preventing adverse CVD events in T2D are inconclusive (Abdul‐Ghani et al., 2017). Participation in effective diabetes self‐care behaviours such as increasing physical activity, healthy dietary patterns and not smoking have been shown to be effective for the reduction of CVD risk in this population (Long, Cooper, Wareham, Griffin, & Simmons, 2014; Wong et al., 2015).

Diabetes self‐care behaviours are important for the improvement of glycaemic control in T2D. Diabetes self‐care behaviours are characterized as the development of expertise that includes transitioning from a passive recipient of care to active participation in disease management (Paterson & Thorne, 2000). Studies have consistently demonstrated that adherence to the recommended diabetes self‐care behaviours is associated with better glycaemic control in T2D (Al‐Khawaldeh, Al‐Hassan, & Froelicher, 2012; Captieux et al., 2018; Kamuhabwa & Charles, 2014; Khattab, Khader, Al‐Khawaldeh, & Ajlouni, 2010; Lee, Piette, Heisler, Janevic, & Rosland, 2019; Zheng, Liu, Liu, & Deng, 2019). A meta‐analysis of the effect of diabetes self‐care interventions identified reduction of glycated haemoglobin (HbA1c) between 0.2%–0.6% at 6 months postintervention in people with T2D (Captieux et al., 2018). A recently published randomized controlled trial of an outpatient diabetes self‐care programme for people with T2D reported that the intervention significantly improved fasting blood glucose (FBG), postprandial blood glucose and HbA1c (Zheng et al., 2019).

Demographic, clinical and psychosocial variables may also influence glycaemic control. Studies have shown that female gender, advancing age, using more than one antiglycaemic agents, lack of health insurance and obesity (Kamuhabwa & Charles, 2014), duration of DM (Kamuhabwa & Charles, 2014; Khattab et al., 2010) and negative attitude towards DM (Khattab et al., 2010) were associated with poor glycaemic control in people with T2D. Higher diabetes self‐efficacy, social support (Shao, Liang, Shi, Wan, & Yu, 2017) and satisfaction with diabetes treatment (Moreira et al., 2010) are associated with better glycaemic control in T2D.

### 1.2 Conceptual framework

This study adapted an integrated conceptual framework originally designed to guide a self‐care intervention in HF (Dunbar, Clark, Quinn, Gary, & Kaslow, 2008). The framework incorporates concepts from self‐management theories and adult learning concepts used in patient education. The variables in the conceptual framework are organized around antecedents and outcomes of self‐care behaviours (Aga, Dunbar, Kebede, Higgins, & Gary, 2019). An individual's demographic, clinical and psychosocial characteristics were considered as antecedents to diabetes self‐care behaviours. The clinical characteristics involve variables depicting duration and severity of both T2D and HF while the psychosocial variables are diabetes self‐efficacy, social support, depression and diabetes knowledge. The diabetes self‐care outcome in this study was glycaemic control indicated by HbA1c and FBG values. To date, most studies that have assessed the relationship between diabetes self‐care behaviours and glycaemic control did not take into account the presence of another serious chronic illness such as HF. As a result, there is little research on how HF may influence diabetes self‐care behaviours and glycaemic control in T2D. Because T2D and HF often are coexisting chronic conditions, a greater understanding of factors that may influence diabetes self‐care behaviours are essential for designing effective interventions that can improve glycaemic control and prevent the occurrence of adverse events. The purpose of this study was to describe the relationship between diabetes self‐care behaviours and glycaemic control (HbA1c and FBG) in adult patient withT2D and comorbid HF. This study, therefore, addressed a main research question: What is the relationship between diabetes self‐care behaviours and glycaemic control in adults with T2D and comorbid HF?

## 2 THE STUDY

### 2.1 Design

This study used a cross‐sectional, correlational design to analyse baseline data from a randomized clinical trial that tested a 6‐month integrated self‐care intervention in adult withT2D and comorbid HF (Dunbar et al., 2014, 2015). The original study aimed at improving HF‐ and T2D‐specific outcomes. The present study explored the relationship between diabetes self‐care behaviours and glycaemic control as measured before intervention was started.

## 3 METHODS

### 3.1 Participants

The parent study enrolled adult patients with comorbid HF and T2D during hospitalization or within 3 months of discharge for worsening HF. Participants’ enrolment was performed at one of four large urban‐tertiary hospitals in the south‐eastern part of the United States from 2010–2013. Participants, who were aged 21–80 years, with New York Heart Association (NYHA) class II‐IV and were either currently hospitalized or recently discharged within the last 3 months, were enrolled in the study. Other inclusion criteria included the presence of T2D, ambulatory and eligible for physical activity, prescribed optimal HF medications according to guidelines and eligible for low sodium and carbohydrate diets. The exclusion criteria were new diagnosis or first HF admission; cognitive impairment score of 11 or above on the Blessed Cognitive Screening Tool; uncorrected hearing or vision problem; undergoing evaluation for cardiac transplant or evaluation for ventricular assist device; renal failure; lack of telephone access; and severe chronic pulmonary disease and previous stroke impeding ambulation and ability to exercise. The study findings are reported in accordance with the Strengthening the Reporting of Observational Studies in Epidemiology (STROBE) checklist for cross‐sectional studies (Vandenbroucke et al., 2014) (Supporting Information).

The sample size was computed using Green's recommendation for the determination of sample size for multiple regression analysis (Green, 1991). A total of 180 participants’ baseline data were included in this study. The effect size (*f* ^2^) for this sample size is 0.04, which is small to medium and satisfied the requirement for a power of 0.80 based on Cohen's recommendation at statistical significance level of 0.05 (Cohen, 1988).

### 3.2 Measurement

#### 3.2.1 Demographic and clinical information

Demographic and clinical data were collected from medical records and self‐report. The variables included age, gender, race/ethnicity, marital status, education, body mass index (BMI), perceived health rating, living arrangement, NYHA functional classification, years since diagnosis of T2D and HF, left ventricular ejection fraction (LVEF), diabetes with end‐organ failure, diabetes management regimen/type and medications. The number of comorbid chronic conditions was documented using the Charlson comorbidity index (Charlson, Pompei, Ales, & MacKenzie, 1987).

#### 3.2.2 Diabetes self‐care behaviours

The Summary of Diabetes Self‐Care Activities (SDSCA) was used to measure the self‐care behaviours of the study participants (Toobert, Hampson, & Glasgow, 2000). The SDSCA has a core set of 11 items used to measure the diabetes self‐care behaviours of diet, exercise, blood glucose testing, foot care and smoking. Participants reported how many days in the previous week they have engaged in a particular self‐care activity and scores were calculated for each dimension. The total score range 0–28 for diet, 0–14 for exercise, 0–14 for self‐monitoring blood glucose and 0–14 for foot care dimension (Toobert et al., 2000). A study reported Cronbach's alpha of 0.76 for the overall scale and 0.89 for diet, 0.83 for exercise, 0.92 for blood glucose testing and 0.77 for foot care dimensions (Aljohani, Kendall, & Snider, 2016).

#### 3.2.3 Glycaemic control

HbA1c and FBG were used to measure glycaemic control. Both biomarkers were analysed from whole blood samples at baseline. HbA1c is the gold standard for monitoring glycaemic control and reflects a person's glucose control for the preceding 3 months (ADA, 2019). FBG provides immediate blood ranges to guide treatment, food and activity choices (Krhac & Lovrencic, 2019). The American Diabetes Association (ADA, 2019) recommends a glycaemic goal of HbA1c < 7% (53 mmol/mol), which equivalent to FBG 80–130 mg/dl (4.4–7.2 mM) for the general population of persons with T2D. Analysis was conducted in the clinical laboratory using high‐performance liquid chromatography, with standardization through commercially available controls (CV < 2%). FBG test was also performed at the clinical laboratory after 8 hr of fasting.

#### 3.2.4 Diabetes self‐efficacy

Diabetes self‐efficacy was measured using the 8‐item perceived diabetes self‐management scale (PDSMS). The scale was developed by Wallston and colleagues (Wallston, Rothman, & Cherrington, 2007) and has responses for each item ranging from 1 (strongly disagree) to 5 (strongly agree) with the total score ranging from 8–40. A higher score shows more confidence in performing diabetes self‐care behaviours. The PDSMS has adequate reported reliability with Cronbach's alpha between 0.83–0.93 (Al‐Aboud, Ahmad, Bidin, & Ismail, 2016).

#### 3.2.5 Diabetes knowledge

The Michigan Diabetes Knowledge Test (DMKT) was used to measure the participants’ knowledge of diabetes (Fitzgerald et al., 2016). The tool was scored based on the per cent of questions answered correctly. The DMKT consists of 14‐item and has adequate reported reliability with a Cronbach's alpha of 0.77 (Fitzgerald et al., 2016).

#### 3.2.6 Depression

The Patient Health Questionnaire‐9 (PHQ‐9) is a widely used depression screening instrument and has been used in T2D and in HF populations (Kroenke, Spitzer, & Williams, 2001). The 9‐item depression scale includes ratings of symptoms as 0 (indicating not at all) to 3 (nearly every day). Scores range from 0–27, with a score of 5–9 reflecting mild depressive symptoms to ≥10 indicating moderate to severe depressive symptoms. The PHQ‐9 has adequate internal reliability with a Cronbach's alpha of 0.89 (Yaung et al., 2008).

#### 3.2.7 Social support

The Enhancing Recovery in Coronary Heart Disease (ENRICHD) Social Support Instrument (ESSI) was used to assess the social support available to the participants (ENRICHD, 2000). The instrument is a 7‐item self‐report survey that assesses perceived social support and has been used with several studies. All items values are summed for a total score, with higher score denoting greater social support. The ESSI has demonstrated acceptable internal consistency with a Cronbach's alpha of 0.86–0.88 (Vaglio et al., 2004).

### 3.3 Data analysis

Prior to analysis, the data were examined for accuracy, completeness, potential outliers and missing values. The distributions of outcome variables (HbA1c and FBG) were scrutinized for normality by visual inspection and the Shapro–Wilk and Kolmogorov–Smirnov tests. The normality assessment revealed skewness in the distribution of these outcome data. This problem was resolved using square root and base 10 log transformation methods. The HbA1c data that had moderately positive skewness was square root transformed while the FBG data that had substantial positive skewness was base 10 log transformed (Tab achnick & Fidell, 2007). Bivariate analysis using correlation coefficients (Pearson's *r* and Spearman's *ρ*), independent‐sample *t* test and one‐way analysis of variance (ANOVA) was conducted to assess the demographic, clinical, psychosocial variables and diabetes self‐care behaviours relationships with HbA1c and FBG. Pearson's *r* was used when the two variables in the correlation analysis were normally distributed, and Spearman's *ρ* was used when the assumption of normality violated by at least one variable in the analysis and when this problem could not be fixed using data transformation method. Statistical significance was set at 5%. Hierarchical multiple regressions were used to assess whether greater diabetes self‐care behaviours were associated with better glycaemic control (lower glycated haemoglobin [HbA1c] and FBG) after controlling for demographic, clinical and psychosocial variables. Multi‐categorical independent variables were dummy coded before entering into the models. To avoid the issues of multicollinearity, inter‐correlations between variables were examined but no inter‐correlation higher than 0.80 was found. Moreover, the Durbin–Watson test was used to assess the independence of residuals (value of 2 for complete independence). Magnitude of difference and effect sizes were also computed for each variable. The data were analysed using SPSS for Windows version 24 (IBM SPSS Statistics 24).

### 3.4 Ethical considerations

The institutional review boards of all partaking institutions approved the study. All participants provided written informed consent and signed Health Insurance Privacy and Portability prior to baseline measures.

## 4 RESULTS

### 4.1 Participant characteristics

The mean age of the study participants was 58 ± 10.7 years (Table 1), most were male (*N* = 118, 66%) and African American (*N* = 119, 66%). Most had more than a high school education (*N* = 114, 63.3%) and lived with a spouse or children (*N* = 116, 64.4%). The mean number of years since diagnosis of T2D was 11 (*SD* 8.4) and 5 (*SD* 5.9) for HF (Table 2). Clinically, the LVEF was 34 (*SD* 16.9); the number of medications taken was 12 (*SD* 3.9), and most had an average of 4 (*SD* 2.3) chronic conditions (Table 3). Before data transformation, the mean HbA1c was 8 ± 1.8% and the mean FBG was 165 (*SD* 79.4) mg/dl (Table 2). The mean score of general dietary self‐care behaviour was 5 ± 1.9, and diabetes‐specific dietary self‐care behaviour was 5 (*SD* 1.5) per week (Table 1). Most participants (*N* = 109, 67%) performed foot care less than 7 days per week while 86 (48%) undertook blood glucose testing for less than 7 days per week and 66 (37%) did not exercise a minimum one day per week. The mean depression score was 7 (*SD* 4.4).

**Table 1:** Demographic characteristics, psychosocial factors and self‐care behaviours of T2D–comorbid HF patients on glycaemic control (*N* = 180)

Characteristics	Overall *N* (%)	Glycated haemoglobin (HbA1c) (Square root transforme*d*)		Fasting blood glucose (Log10 transformed)	
		Mean ± *SD*	Test of association	Mean ± *SD*	Test of association
Demographic					
Age, years, mean ± *SD*	58.1 ± 10.7	2.8 ± 0.3	*r* = −.17, *p* = .03	2.2 ± 0.2	*r* = −.129, *p* = .094
Sex					
Female	62 (34.4%)	2.9 ± 0.3	*t* = 0.599, *p* = .551	2.1 ± 0.2	*t* = −0.470, *p* = .639
Male	118 (65.6%)	2.8 ± 0.3		2.2 ± 0.2	
Race					
None‐AA (white or Asian)	61 (33.9%)	2.8 ± 0.2	*t* = 0.277, *p* = .782	2.2 ± 0.2	*t* = 0.474, *p* = .636
African American (AA)	119 (66.1%)	2.8 ± 0.3		2.2 ± 0.2	
Marital status					
Single/divorced/widowed	90 (50%)	2.8 ± 0.3	*t* = 0.035, *p* = .973	2.2 ± 0.2	*t* = 0.303, *p* = .762
Married/domestic partner	90 (50%)	2.8 ± 0.3		2.2 ± 0.2	
Education					
≤High school	65 (36.1%)	2.9 ± 0.3	*t* = 0.945, *p* = .347	2.2 ± 0.2	*t* = 0.596, *p* = .552
>High school	114 (63.3%)	2.8 ± 0.3		2.2 ± 0.2	
Perceived health rating					
Poor	53 (29.4%)	2.8 ± 0.3	1‐ANOVA = *F* (2, 148) = 0.882, *p* = .416	2.1 ± 0.2	1‐ANOVA = *F* (92, 161) = 0.803, *p* = .450
Fair	73 (40.6%)	2.9 ± 0.3		2.2 ± 0.2	
Good	49 (27.2%)	2.8 ± 0.3		2.2 ± 0.2	
BMI, kg/m2, mean ± *SD*	36.7 ± 8.9	2.8 ± 0.3	*ρ* = 0.115, *p* = .153	2.2 ± 0.2	*ρ* = 0.127, *p* = .099
Living arrangement					
Live alone	41 (22.8%)	2.8 ± 0.3	1‐ANOVA = *F* (2, 153) = 0.241, *p* = .786	2.2 ± 0.2	1‐ANOVA = *F* (2, 166) = 0.372, *p* = .690
Live with spouse/children	116 (64.4%)	2.8 ± 0.3		2.2 ± 0.2	
Live with siblings/other relatives	23 (12.8%)	2.8 ± 0.2		2.1 ± 0.2	
Psychosocial factors					
Depression, mean ± *SD*	6.7 ± 4.4	2.8 ± 0.3	*ρ* = −0.004, *p* = .960	2.1 ± 0.2	*ρ* = 0.020, *p* = .798
Social support, mean ± *SD*	27.9 ± 5.8	2.8 ± 0.3	*ρ* = 0.020, *p* = .841	2.2 ± 0.2	*ρ* = −0.063, *p* = .509
Self‐efficacy, mean ± *SD*	27.7 ± 6.2	2.8 ± 0.3	*r* = −.231, *p* = .005	2.2 ± 0.2	*r* = −.136, *p* = .084
Diabetes knowledge, mean ± *SD*	57.9 ± 18.2	2.8 ± 0.3	*r* = .024, *p* = .763	2.2 ± 0.2	*r* = .057, *p* = .463
Diabetes self‐care behaviours					
General diet, mean ± *SD*	5.1 ± 1.9	2.8 ± 0.3	*r* = −.171, *p* = .036	2.2 ± 0.2	*r* = −.068, *p* = .392
Specific diet. mean ± *SD*	4.6 ± 1.5	2.8 ± 0.3	*r* = .162, *p* = .047	2.2 ± 0.2	*r* = −.095, *p* = .223
Exercise					
=0 days per week	66 (36.7%)	2.8 ± 0.3	*t* = 2.208, *p* = .029	2.2 ± 0.2	*t* = 0.400, *p* = .690
>0 days per week	109 (60.6%)	2.8 ± 0.3		2.2 ± 0.2	
Blood glucose testing					
< 7 days per week	86 (47.8%)	2.8 ± 0.4	*t* = −0.045, *p* = .579	2.2 ± 0.2	*t* = 1.565, *p* = .120
= 7 days per week	88 (48.9%	2.8 ± 0.2		2.1 ± 0.2	
Foot care					
<7 days per week	109 (60.6%)	2.8 ± 0.3	*t* = 0.895, *p* = .373	2.2 ± 0.2	*t* = 0.654, *p* = .514
=7 days per week	66 (36.7%)	2.9 ± 0.3		2.1 ± 0.2	

Abbreviations: 1‐ANOVA, One‐way analysis of variance; *r*, Pearson's correlation coefficient; *t*, Independent sample *t* test; *ρ*, Spearman's correlation coefficient.

**Table 2:** Clinical characteristics of T2D–comorbid HF patients on glycemic control (*N* = 180)

Characteristics	Overall *N* (%)	Glycated haemoglobin (HbA1c) (Square root transformed)		Fasting blood glucose (Log10 transformed)	
		Mean ± *SD*	Test of association	Mean ± *SD*	Test of association
Glycated haemoglobin (HbA1c)	8.1 ± 1.8%				
Fasting blood glucose (FBG)	165 ± 79.4 mg/dl				
NYHA Functional class					
Class I and II	70 (38.9%)	2.9 ± 0.3	*t* = 1.525, *p* = .130	2.2 ± 0.2	*t* = −0.032, *p* = .975
Class III and IV	109 (60.6%)	2.8 ± 0.3		2.2 ± 0.2	
HF duration, y, mean ± *SD*	5.3 ± 5.9	2.8 ± 0.3	*ρ* = −0.075, *p* = .362	2.2 ± 0.2	*ρ* = −0.202, *p* = .009
Ejection fraction, mean ± *SD*	33.6 ± 16.9	2.8 ± 0.3	*ρ* = 0.061, *p* = .452	2.2 ± 0.2	*ρ* = 0.062, *p* = .427
DM duration, y, mean ± *SD*	11.1 ± 8.4	2.8 ± 0.3	*ρ* = 0.076, *p* = .345	2.2 ± 0.2	*ρ* = 0.046, *p* = .558
DM with end‐organ failure					
No	137 (76.1%	2.8 ± 0.3	*t* = −1.012, *p* = .315	2.1 ± 0.2	*t* = −1.250, *p* = .216
Yes	42 (23.3%)	2.9 ± 0.3		2.2 ± 0.2	
DM management regimen					
Diet alone	15 (8.3%)	2.5 ± 0.2	*t* = −5.694, *p* = .000	2.1 ± 0.2	*t* = −2.026, *p* = .059
Diet plus medications	163 (90.6%)	2.9 ± 0.3		2.2 ± 0.2	
ACE inhibitor use					
No	81 (45.0%)	2.8 ± 0.3	*t* = −1.577, *p* = .117	2.1 ± 0.2	*t* = −1.351, *p* = .179
Yes	97 (53.9%)	2.8 ± 0.3		2.2 ± 0.2	
ARB use					
No	146 (81.1%	2.8 ± 0.3	*t* = 1.302, *p* = .201	2.2 ± 02	*t* = −0.443, *p* = .662
Yes	25 (13.9%)	2.8 ± 0.2		2.2 ± 0.2	
Beta blockers use					
No	11 (6.1%)	2.9 ± 0.3	*t* = 0.360, *p* = .728	2.2 ± 0.3	*t* = −0.099, *p* = .923
Yes	167 (92.8%)	2.8 ± 0.3		2.2 ± 0.2	
Diuretics use					
No	127 (70.6%)	2.8 ± 0.3	*t* = −0.791, *p* = .432	2.1 ± 0.2	*t* = −1.716, *p* = .091
Yes	45 (25.0%)	2.9 ± 0.3		2.2 ± 0.2	
Loop diuretics use					
No	21 (11.7%)	2.9 ± 0.4	*t* = 0.413, *p* = .684	2.1 ± 02	*t* = −0.758, *p* = .456
Yes	158 (87.8%)	2.8 ± 0.3		2.2 ± 0.2	
Aldosterone inhibitors use					
No	113 (62.8%)	2.8 ± 0.3	*t* = −0.656, *p* = .514	2.2 ± 0.2	*t* = 1.175, *p* = .243
Yes	49 (27.2%)	2.9 ± 0.3		2.1 ± 0.2	
Digitalis use					
No	156 (86.7%)	2.8 ± 0.3	*t* = 0.423, *p* = .677	2.2 ± 0.2	*t* = 1.996, *p* = .058
Yes	20 (11.1%)	2.8 ± 0.3		2.1 ± 0.2	
Total daily medication, mean ± *SD*	12.2 (3.9)	2.8 ± 0.3	*ρ* = −0.103, *p* = .200	2.2 ± 0.2	*ρ* = 0.009, *p* = .906
Oral DM medication use					
No, unknown or missing	114 (63.3%)	2.9 ± 0.3	*t* = 1.372, *p* = .172	2.2 ± 0.2	*t* = 0.823, *p* = .412
Yes	66 (36.7%)	2.8 ± 0.2		2.1 ± 0.2	
Insulin use					
No, unknown or missing	66 (36.7%)	2.7 ± 0.2	*t* = −6.297, *p* = .000	2.1 ± 0.2	*t* = −3.214, *p* = .002
Yes	114 (63.3%)	2.9 ± 0.3		2.2 ± 0.2	
DM medication categorys					
Oral medication only	48 (26.7%)	2.7 ± 0.2	1‐ANOVA = *F* (3, 152) = 16.250, *p* = .000; SDP: No to both vs Oral med only^a^, No to both vs Insulin only^b^, No to both vs Yes to both^c^	2.1 ± 0.2	1‐ANOVA = *F* (3, 165) = 3.744, *p* = .012; SDP: No to both Insulin only: MD = 0.17, *p* = .015
Insulin only	96 (53.3%)	2.9 ± 0.3		2.2 ± 0.2	
Both oral and insulin	18 (10.0%)	2.9 ± 0.2		2.2 ± 0.2	
No to both	18 (10.0%)	2.5 ± 0.2		2.0 ± 0.1	

Abbreviations: 1‐ANOVA, One‐way analysis of variance; SDP, Sidak post hoc test; *t*, Independent sample *t* test; *ρ*, Spearman's correlation coefficient.

^a^ Mean difference (MD) = 0.26 (*p* = .007).

^b^ MD = 0.46 (*p* < .001).

^c^ MD = 0.45 (*p* < .001).

**Table 3:** Multimorbid conditions on glycemic control of T2D–comorbid HF patients (*N* = 180)

Characteristics	Overall *N* (%)	Glycated haemoglobin (HbA1c) (Square root transformed)		Fasting blood glucose (Log10 transformed)	
		Mean ± *SD*	Test of association	Mean ± *SD*	Test of association
CCI, mean ± *SD*	4.2 ± 2.3	2.8 ± 0.3	*ρ* = −0.091, *p* = .256	2.2 ± 0.2	*ρ* = −0.029, *p* = .704
Charlson comorbidity > 2					
No	47 (26.1%)	2.9 ± 0.3	*t* = 2.657, *p* = .010	2.2 ± 0.2	*t* = 0.850, *p* = .398
Yes	133 (73.9%)	2.8 ± 0.3		2.2 ± 0.2	
Depression history					
No	136 (75.6%)	2.8 ± 0.3	*t* = 0.545, *p* = .588	2.2 ± 0.2	*t* = −0.963, *p* = .340
Yes	41 (22.8%)	2.8 ± 0.3		2.2 ± 0.2	
Hypertension					
No	9 (5.0%)	2.9 ± 0.3	*t* = 0.292, *p* = .777	2.2 ± 0.1	*t* = 1.614, *p* = .142
Yes	170 (94.4%)	2.8 ± 0.3		2.2 ± 0.2	
Arthritis					
No	135 (75.0%)	2.9 ± 0.3	*t* = 2.923, *p* = .004	2.2 ± 02	*t* = 1.055, *p* = .295
Yes	43 (23.9%)	2.7 ± 0.2		2.1 ± 0.2	
CABG					
No	142 (78.9%)	2.8 ± 0.3	*t* = 331, *p* = .742	2.2 ± 0.2	*t* = 0.005, *p* = .996
Yes	38 (21.1%)	2.8 ± 0.3		2.2 ± 0.2	
History of valve repair					
No	170 (94.4%)	2.8 ± 0.3	*t* = 1.959, *p* = .084	2.2 ± 0.2	*t* = 4.480, *p* = .001
Yes	8 (4.4%)	2.7 ± 0.2		2.0 ± 0.1	
History of valve replacement					
No	171 (95.0%)	2.8 ± 0.3	*t* = 0.394, *p* = .705	2.2 ± 0.2	*t* = 0.361, *p* = .726
Yes	8 (4.4%)	2.8 ± 0.3		2.1 ± 0.1	
History of PTCA or PCI					
No	128 (71.1%)	2.8 ± 0.3	*t* = 0.123, *p* = .902	2.2 ± 0.2	*t* = 1.495, *p* = .138
Yes	50 (27.8%)	2.8 ± 0.2		2.1 ± 0.2	
History of stent					
No	140 (77.8%)	2.8 ± 0.3	*t* = −0.386, *p* = .700	2.2 ± 0.2	*t* = −0.579, *p* = .565
Yes	39 (21.7%)	2.8 ± 0.2		2.2 ± 0.2	
Dyslipidemia					
No	83 (46.1%)	2.8 ± 0.3	*t* = 0.957, *p* = .341	2.1 ± 0.2	*t* = −0.559, *p* = .577
Yes	94 (52.2%)	2.8 ± 0.2		2.2 ± 0.2	
Sleep apnoea					
No	115 (63.9%)	2.8 ± 0.3	*t* = 0.432, *p* = .666	2.2 ± 0.2	*t* = 0.488, *p* = .626
Yes	64 (36.6%)	2.8 ± 0.3		2.2 ± 0.2	
Thyroid disorder					
No	158 (87.8%)	2.8 ± 0.3	*t* = 0.509, *p* = .615	2.2 ± 0.2	*t* = 1.750, *p* = .093
Yes	21 (11.7%)	2.8 ± 0.3		2.1 ± 0.2	
Exposure to chemotherapy					
No	172 (96.6%)	2.8 ± 0.3	*t* = −0.571, *p* = .591	2.2 ± 0.2	*t* = 0.874, *p* = .427
Yes	5 (2.8%)	2.9 ± 0.1		2.1 ± 0.2	
Pacemaker or devise					
No	152 (84.4%)	2.8 ± 0.3	*t* = 0.111, *p* = .912	2.2 ± 0.2	*t* = −1.129, *p* = .269
Yes	27 (15.0%)	2.8 ± 0.3		2.2 ± 0.2	
ICD					
No	105 (58.3%)	2.9 ± 0.3	*t* = 1.280, *p* = .203	2.2 ± 0.2	*t* = −0.159, *p* = .874
Yes	72 (40.0%)	2.8 ± 0.3		2.2 ± 0.2	

Abbreviations: CCI, Charlson Comorbidity Index; *t* = Independent sample *t* test; *ρ*, Spearman's correlation coefficient.

### 4.2 Demographic, psychosocial and diabetes self‐care behaviours relationship with glycaemic control

The bivariate analysis (Table 1) showed significant negative correlations between HbA1c and age (*r* = −.17, *p* = .03), diabetes self‐efficacy (*r* = −.231, *p* = .005) and general dietary self‐care behaviour (*r* = −.171, *p* = .036). An increase in age, diabetes self‐efficacy and general dietary self‐care behavior were associated with a decrease in HbA1c. Specific dietary self‐care behaviour had a significant positive correlation with HbA1c (*r* = .162, *p* = .047). The independent‐sample *t* test revealed that participants who did not exercise for a minimum of 1 day per week compared with those who exercised 1 day or more per week had a significantly higher HbA1c value, *t* (150) = 2.208, *p* = .029 (Mean difference [MD] = 0.11, 95% CI: 0.011, 0.22).

### 4.3 Clinical characteristics relationship with glycaemic control

An independent‐sample *t* test (Table 2) revealed that participants who were managed with diet plus medication compared with those who managed with diet alone had significant lower HbA1c values, *t* (153) = −5.694, *p* < .001 (MD = −0.32, 95% CI: −0.44, −0.20). Similarly, participants who were using insulin compared with those who were not using insulin had significantly lower HbA1c values, *t* (154) = −6.297, *p* < .001 (MD = −0.27, 95% CI: −0.35, −0.19).

A one‐way between groups ANOVA was conducted to explore the impact of medication categories on HbA1c level (Table 2). Participants were divided into four diabetes medication categories: oral diabetes medication only, insulin only, both oral diabetes medication and insulin and no to both oral diabetes medication and insulin. There was a significant difference (*p* < .05) level in HbA1c values for the four diabetes medication categories, *F* (3, 152) = 16.250, *p* < .001. The difference in mean scores of HbA1c between the diabetes medication categories was quite large with a partial eta square (effect size) of 0.24. Post hoc comparison using Sidak test indicated there were significantly higher HbA1c values among participants who were not taking both oral diabetes medication and insulin compared with those who were taking oral diabetes medication only (MD = 0.26, 95% CI: 0.04, 0.46), taking insulin only (MD = 0.46, 95% CI: 0.26, 0.65) and taking both oral diabetes medication and insulin (MD = 0.45, 95% CI: 0.20, 0.69).

Correlation analysis (Table 2) showed significant inverse relationships between years since diagnosis of HF and FBG level (*ρ* = −0.202, *p* = .009). An independent‐sample *t* test revealed that participants who were using insulin compared with those who were not using insulin had statistically significant lower FBG level, *t* (167) = −3.214, *p* = .002 (MD = −0.10, 95% CI: −0.15, −0.03). A one‐way between groups ANOVA also identified that there was significant difference (*p* < .05) in FBG values for the four diabetes medication categories, *F* (3, 165) = 3.744, *p* = .012. The actual difference in mean scores of FBG between the diabetes medication categories was medium with a partial eta square of 0.06. Post hoc comparison using Sidak test indicated that there were statistically significant higher FBG values among participants who were not taking both oral diabetes medication and insulin compared with those who taking insulin only (MD = 0.17, 95% CI: 0.02, 0.31).

### 4.4 Multi‐morbidity and glycaemic control

The independent‐sample *t* test (Table 3) revealed that participants having more than two comorbid conditions (Charlson comorbidity > 2) compared with those with only two comorbid conditions (T2D and HF only) had a significantly higher HbA1c level (Table 3), *t* (154) = 2.657, *p* = .01. There were several disease‐specific conditions that influenced HbA1c values. Participants who had arthritis compared with those without arthritis had higher HbA1c values, *t* (152) = 2.923, *p* = .004 (MD = 0.13, 95% CI: 0.04, 0.22). Compared with participants without a history of valve repair to those who had history of valve repair had statistically significant higher FBG level, *t* (165) = 4.480, *p* = .001 (MD = 0.13, 95% CI: 0.06, 0.19).

### 4.5 Predictors of glycaemic control

Hierarchical linear regression analysis was used to assess whether greater diabetes self‐care behaviours were associated with better glycaemic control (HbA1c and FBG) after controlling for demographic, clinical and psychosocial variables. Demographic variables were entered at Step 1 (Model 1), clinical variables added at Step 2, Charlson comorbidity index scores were added at Step 3, psychosocial variables added at Step 4 and diabetes self‐care behaviours added at Step 5. None of the specific diabetes self‐care behaviours emerged as a predictor of HbA1c or FBG (Tables 4 and 5).

**Table 4:** Summary of hierarchical regression analysis for variables predicting HbA1c (*N* = 180)

Variable	Model 1			Model 2			Model 3			Model 4			Model 5		
	*B*	*SE B*	*β*	*B*	*SE B*	*β*	*B*	*SE B*	*β*	*B*	*SE B*	*β*	*B*	*SE B*	*β*
DM with end‐organ failure (Yes)				0.248	0.90	0.337**	0.292	0.125	0.397*	0.277	0.135	0.376*	0.304	0.138	0.413*
DM medication categories:															
Take insulin only				0.250	0.087	0.409**	0.247	0.096	0.404*	0.267	0.261	−0.427*	0.243	0.113	0.396*
Taking both oral med and insulin				0.391	0.134	0.393**	0.356	0.148	0.357*	0.367	0.158	0.369*	0.344	0.165	0.345*
African American race							0.259	0.098	0.398*	0.242	0.105	0.372*	0.304	0.145	0.466*
Dyslipidemia							0.197	0.093	0.320*	0.214	0.101	0.348*	0.296	0.123	0.481*
*R* ^2^	0.106			0.516			0.694			0.699			0.745		
*F* for change in *R* ^2^	0.787			2.788**			1.460			0.138			1.133		

Abbreviation: *SE B*, Standard error of *B* (unstandardized regression coefficient).

* *p* < .05;

** *p* < .01.

**Table 5:** Summary of hierarchical regression analysis for variables predicting FBG (*N* = 180)

Variable	Model 1			Model 2			Model 3			Mode 4		
	*B*	*SE B*	*β*	*B*	*SE B*	*β*	*B*	*SE B*	*β*	*B*	*SE B*	*β*
HF duration (years)				−0.011	0.004	−0.284*	−0.017	0.004	−0.461***	−0.015	0.005	−0.416**
DM medication categories:												
Take insulin only				0.118	0.056	0.302*	0.160	0.061	0.410*	0.197	0.066	0.505**
Take both oral med and insulin				0.161	0.088	0.245	0.234	0.092	0.357*	0.238	0.095	0.362*
LVEF							−0.004	0.002	−0.343*	−0.004	0.002	−0.360*
DM with end‐organ failure							0.218	0.077	0.453**	0.219	0.078	0.454**
Total daily medications							0.016	0.007	0.301*	0.018	0.008	0.354*
Exposure to chemotherapy							−0.274	0.135	−0.249*	−0.323	0.140	−0.294*
*R* ^2^	0.088			0.435			0.657			0.679		
*F* for change in *R* ^2^	0.712			2.315**			1.934*			0.775		

Abbreviation: LVEF, Left ventricular ejection fraction.

* *p* < .05;

** *p* < .01;

*** *p* < .001.

As presented in Table 4, none of the variables entered in Model 1 were significant predictors of HbA1c. However, T2D with end‐organ failure and the two diabetes medication categories (taking insulin only and taking both oral diabetes medications and insulin) emerged as significant predictors of HbA1c in Model 2 (Table 4). Model 2 explained 51.6% of the variance in HbA1c, *F* (28, 56) = 2.131, *p* = .008. In Model 3, African American race and dyslipidaemia emerged significant predictors of HbA1c and these remained in Model 4 and 5. Model 3 explained 69.4% [*F* (44, 40) = 2.065, *p* = .011] and Model 4 explained 69.9% [*F* (48, 36) = 1.741, *p* = .043] of the variance in HbA1c. Model 5 also explained 74.5% of the variance in HbA1c but this model was not significant, *F* (53, 31) = 1.713, *p* = .055. In the final model (Model 5), all the five variables were statistically significant, with dyslipidaemia having a higher beta value (*β* = 0.481, *p* < .05).

As presented in Table 5, none of the variables entered in Model 1 were significant predictors of FBG. HF duration (years) and the two diabetes medication categories (taking insulin only and taking both oral diabetes medications and insulin) emerged as significant predictors of FBG in Model 2 (Table 5). Model 2 explained 43.5% of the variance in FBG, *F* (28, 64) = 1.762, *p* = .032. In Model 3, LVEF, diabetes with end‐organ failure, total daily medications and exposure to chemotherapy emerged as significant predictors of FBG and these remained in Mode 4. Model 3 explained 65.7% [*F*(44, 48) = 2.087, *p* = .007] while Model 4 explained 67.9% [*F*(48, 44) = 1.942, *p* = .014], with taking insulin only having a higher beta value (*β* = 0.505, *p* < .01).

The condition indexes are <30, and the variance inflation factors (VIF) are <5, which supports that multicollinearity was not an issue for the predictor variables in Tables 4 and 5. The assumption of independence of residuals was consistent with a Durbin–Watson test value of <2 for the regression models of HbA1c and FBG.

## 5 DISCUSSION

The relationships between diabetes self‐care behaviours and glycaemic control in persons with T2D and comorbid HF have not been previously reported. The major finding was that T2D‐HF patients with greater adherence to the SDSCA general diet and exercise recommendations had better glycaemic control, corroborating previous studies (Cavero‐Redondo et al., 2018; Sainsbury et al., 2018). However, contrary to previous findings (Sainsbury et al., 2018), participants who adhered to the SDSCA specific diet had higher HbA1c levels. In addition, findings showed that persons with greater than two comorbidities had poorer glycaemic control which suggests that greater disease burden has a negative impact on an individual's ability to perform effective diabetes self‐care behaviours. Likewise, participants were prescribed an average of 12 medications which may also have contributed to higher disease burden and poor diabetes self‐care given the complexity of T2D and HF treatment regimens.

Unlike a previous study in T2D (Kamuhabwa & Charles, 2014), the present study showed that advancing age is associated with decreasing HbA1c level. This may be linked to the neurohormonal perturbation that occur in HF (Greene & Felker, 2018) an issue which need to be explored in future research. An unexpected finding was that none of the diabetes self‐care behaviours were predictors of HbA1c or FBG which rejected our hypothesis. These findings provide compelling evidence that clinicians need to shift away from the conventional disease‐specific model of diabetes self‐care to an integrative approach that addresses the complex needs of T2D and HF, medication burden, as well as other comorbidities.

The only demographic variable that predicted higherHbA1c was African American race which is consistent with other studies (Assari, Lankarani, Piette, & Aikens, 2017; Egede, Mueller, Echols, & Gebregziabher, 2010; Kirk et al., 2006). According to the Centers for Disease Control (CDC), approximately 15million men in the US have a diabetes diagnosis, with African American and Hispanic men having a higher prevalence than non‐Hispanic White men (CDC, 2017). African Americans are also reported to have poorer clinical outcomes, more likely to develop complications such as retinopathy and nephropathy and are more likely to be hospitalized with diabetes‐related events associated with T2D in addition to higher risk for mortality (Bell et al., 2010; Davis‐Smith, 2007; Dodani & Fields, 2010; Gatwood et al., 2018). The reasons that African American men have poorer clinical outcomes is not well described (Wessells, 2010). Previous research suggests that higher HbA1c among African Americans with T2D may be associated with socioeconomic status, lack of insurance coverage and lower educational level and poorer self‐care practices (Assari et al., 2017; Egede et al., 2010). Future studies examining culturally appropriate diabetes self‐care interventions are warranted given the high morbidity and poor health outcomes especially among African American's men.

The clinical variables that predicted higher HbA1c were dyslipidaemia, having diabetic end‐organ failure, taking insulin medication only and taking both oral medication and insulin which in part may be a proxy for greater T2D and HF disease severity. Dyslipidaemia was an independent predictor of higher HbA1c which is supported in previous studies (Chandra & Shukla, 2016; Thambiah et al., 2016). Both dyslipidaemia and HbA1c are important risk factors for the development of CVD and higher mortality (Cavero‐Redondo, Peleteiro, Alvarez‐Bueno, Rodriguez‐Artalejo, & Martinez‐Vizcaino, 2017; Lee et al., 2017). Studies suggest that close monitoring and management of dyslipidaemia with lipid‐lowering therapy reduces CVD morbidity and mortality and improves survival (Scicali et al., 2018). Because persons with T2D and HF often have dyslipidaemia as a comorbid condition, it is important they are educated and made aware that adhering to the prescribed lipid‐lowering therapy reduces their risk for developing progressive peripheral arterial disease and the associated vascular complications. Integrated management of hyperglycaemia and dyslipidaemia in person's with T2D and comorbid HF is an important strategy for reducing the risk for adverse CVD and peripheral arterial events and improved glycaemic control (Eeg‐Olofssaon et al., 2016; Hanefeld, Traylor, Gao, & Landgraf, 2017).

The association between the two DM medication categories (taking insulin only and taking both oral medication and insulin) and higher HbA1c and FBG in this study may be due to exacerbation of insulin resistance by the presence of comorbid HF in T2D. Greater T2D disease severity may also play a role poorer glycaemic control. Insulin resistance is common in persons with HF and may have contributed to higher glycaemic values (Doehner, Frenneaux, & Nker, 2014). One study reported that comorbid HF more than doubles the incidence of T2D due to insulin resistance (Guglin, Lynch, & Krischer, 2014). Impaired response to either endogenous or exogenous insulin is a characteristics of insulin resistance (Church & Haines, 2016). This supports the need to look for ways to improve insulin uptake in persons with T2D and comorbid HF. The recommendation to augment drug treatment with self‐care interventions including reduction in caloric intake and increasing exercise could also be useful to improve insulin resistance and to achieve better glycaemic control in this population (Aroor, Mandavia, & Sowers, 2012).

The present study showed that number of years since diagnosis of HF was negatively associated with FBG. Longer duration of HF likely expose persons with T2D to neurohormonal perturbations and increased mortality (Greene & Felker, 2018). A previous study has reported that low FBG and increased glucagon are robust predictors of adverse events, primarily mortality, in patients with advanced HF (Melenovsky et al., 2017). Close FBG monitoring may be a useful intervention for preventing hypoglycaemia and the adverse events associated with it in this population.

This study also revealed that T2D‐HF patients with diabetes‐related end‐organ failure predicted poor control of bothHbA1c and FBG. Previous studies have shown that chronic kidney disease (CKD) is an independent predictor of hypoglycaemia, which is in turn associated with increased risk of mortality in T2D (Chu et al., 2017; Moen et al., 2009). The modifiable risk factors for CKD include poor glycaemic control, hypertension, dyslipidaemia, smoking, low‐grade inflammation, advanced glycation end products, physical inactivity and salt intake (Harjutsalo & Groop, 2014). Targeting these risk factors is needed for the effective prevention and management of CKD in T2D with comorbid HF (Chadban et al., 2010). Emphasis placed on effective glycaemic control, the use of antihypertensive and lipid‐lowering therapies and lifestyle modifications including smoking cessation, diet and physical activity for the prevention and management of CKD in people with T2D and comorbid HF.

The total daily medication was an independent predictor of impaired FBG in the present study. Given that T2D‐HF patients in our study were taking on average 12 or more medications per day, the burden associated with the medication regimen likely has an impact on treatment adherence which can lead to poorer glycaemic control (Bluher, Kurz, Dannenmaier, & Dworak, 2015; Saundankar et al., 2016). The medication burden may be related to the high number of comorbidities observed in our study participants. The use of the fixed‐dose combination drugs to treat common comorbid conditions (Moore et al., 2018) may need to be considered to reduce medication burden and improve FBG in people with T2D and comorbid HF.

Our study also revealed that exposure to chemotherapeutic agents predicts lower FBG level in adults with T2D and HF. Basically, chemotherapy may induce either lower or higher blood glucose level. Previous studies have shown that chemotherapeutic agents such as 6‐meracaptopurine lower blood glucose level (Cho, Moon, Lee, & Ko, 2018) while agents including tyrosine kinase inhibitors induce hyperglycaemia (Goldman, Mendenhau, & Rettinger, 2016). These blood glucose disturbances can result from the side effects of chemotherapeutic agents. Thus, frequent blood glucose monitoring and providing patients with appropriate calories may help to prevent and manage glycaemic fluctuation in this patient population.

### 5.1 Implication for practice and research

The conventional approach for diabetes self‐care intervention is mainly diseases‐specific, overlooking the role of the other comorbid condition(s). Experts in the area have strongly suggested an integrated approach to improve self‐care behaviours and outcomes in persons with comorbid conditions (Dunbar et al., 2014; Smith, Soubhi, Fortin, Hudon, & O'Dowd, 2012). The findings of the present study underscore the importance for clinicians to replace the conventional disease‐specific model of diabetes self‐care with an integrated intervention model that helps to tackle the complex clinical problems of T2D and HF, medication burden and other comorbidities. For example, integrated diabetes self‐care intervention could help to reduce risk factors for CVD and CKD in people with T2D and comorbid HF. Further study is, however, needed to test the effect of an integrated diabetes self‐care intervention on glycaemic control and other patient outcomes. Future studies are also needed to ascertain the underlying reasons for African American men having poor diabetes control outcomes and implement culturally appropriate diabetes self‐care intervention in this population group.

### 5.2 Strength and limitations

There were several strengths of this study. Examining the influence of HF on T2D self‐care behaviours has not been previously reported. The study included a population of predominately African American males who have received less attention regarding T2D self‐care behaviours. The findings from this study add to knowledge about self‐care behaviours in this demographic group. The sample was recruited from four urban, academic tertiary care centres with specialized HF clinics where guidelines for HF are used. The limitations include less generalizability to the larger general community‐based population of patients with T2D and comorbid HF since the participants in the parent study were recruited based on being hospitalized for HF first with a diagnosis of T2D. Another limitation was the cross‐sectional nature of the study design which prevented examination of temporal associations over time. Although confounders were included in our statistical analyses, it is possible that some bias may have occurred, and findings should be interpreted with caution. As with any secondary analysis, we were limited by measures selected in the parent study.

## 6 CONCLUSION

An integrated self‐care framework was used to explore the association between diabetes self‐care behaviours and glycaemic control in people with T2D and comorbid HF. Although none of the diabetes self‐care behaviours independently predicted glycaemic control, some demographic and clinical variables emerged as independent predictors. Many of the clinical variables such as medication regimens that included insulin or combination therapy, number of comorbidities and number of daily medications may be a proxy for more progressive disease and higher disease burden resulting in poorer glycaemic control. The findings suggest that the presence of other comorbidities may heighten the complexity of performing diabetes self‐care, leading to poorer glycaemic control. An unanticipated finding was that none of the diabetes self‐care behaviours influenced glycaemic control. This suggests that the burden associated with having multiple chronic conditions may be an important factor to consider when evaluating the ability to perform effective diabetes self‐care in persons with T2D and comorbid HF. Findings from this study indicate that future research should target multi‐morbidity and how to best manage these complex self‐care and treatment regimens to improve health outcomes and quality of life.

## CONFLICT OF INTEREST

Authors have no conflict of interest.

## AUTHOR CONTRIBUTIONS

FA made substantial contribution to conception and design of the study, data acquisition, analysis and interpretation and drafting the manuscript. RG made substantial contribution to interpretation and critically reviewed and shaped up the drafted manuscript. SBD and TK substantially contributed to the conception and design of the study and critically reviewed the manuscript. MKH contributed to data acquisition, analysis and interpretation. All authors gave final approval of the manuscript and agreed to be accountable for all aspect of the work.

## Supporting information

Supplementary Material
